# Supplementary material for: Spermidine and 1,3-Diaminopropane Have Opposite Effects on the Final Stage of Cephalosporin C Biosynthesis in High-Yielding Acremonium chrysogenum Strain
Source: Int J Mol Sci. 2022 Nov 23;23(23):14625. doi: 10.3390/ijms232314625 (PMC9738377; doi:10.3390/ijms232314625)
Supplement: Supplementary file 1 [file ijms-23-14625-s001.zip › Description of Supplementary Materials.pdf]

Supplementary Materials:

Figure S1: Growth of *A. chrysogenum* wild-type (WT) and high-yielding (HY) strains on agarized Czapek-N medium, supplemented with $NaNO_3$, or NH_4Cl, or urea, or L-Asn (L-asparagine), or L-Gln (L-glutamine) as sole nitrogen source at the concentration of 50 mM. Incubation for 20 days at 26 °C. Scale bar = 2 μm;

Figure S2: Growth phenotype of *A. chrysogenum* wild-type (WT) and high-yielding (HY) strains on agarized Czapek-N agar medium N medium, supplemented with $NaNO_3$, or NH_4Cl, or urea, or L-asparagine, or L-glutamine as sole nitrogen source at the concentration of 1 mM, or 10 mM, or 50 mM, or 100 mM after incubation for 20 days at 26 °C;

Figure S3: Expression dynamics of: (**a**) *cefP*; (**b**) *cefM*; (**c**) *cefT* genes in *A. chrysogenum* HY strain after the addition 5 mM 1,3-diaminopropane (DAP), or 5 mM spermidine (SPD). After 1, 24, 48, 72, 96, 120, and 144 h of fermentation on complex (CP) medium. Data are means ± SD, n=3. Statistical significance, *$p \leq 0.05$, as compared with the control (strain, cultivated on medium without PAs additions).
