# Supplementary figures and images for: Spermidine and 1,3-Diaminopropane Have Opposite Effects on the Final Stage of Cephalosporin C Biosynthesis in High-Yielding Acremonium chrysogenum Strain
Source: Int J Mol Sci. 2022 Nov 23;23(23):14625. doi: 10.3390/ijms232314625 (PMC9738377; doi:10.3390/ijms232314625)

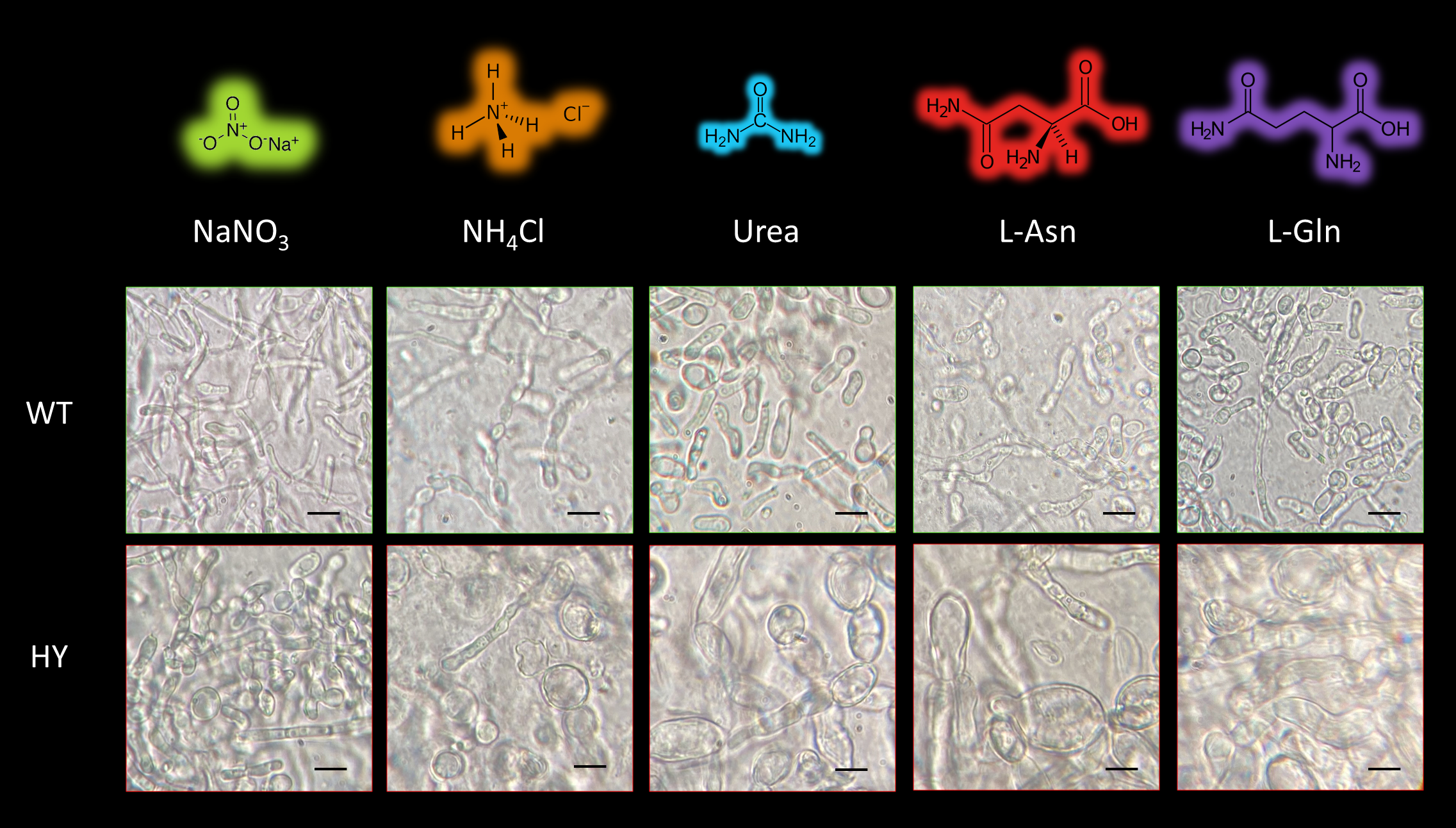

Supplement: Supplementary file 1 [file ijms-23-14625-s001.zip › S1 Figure.tif]

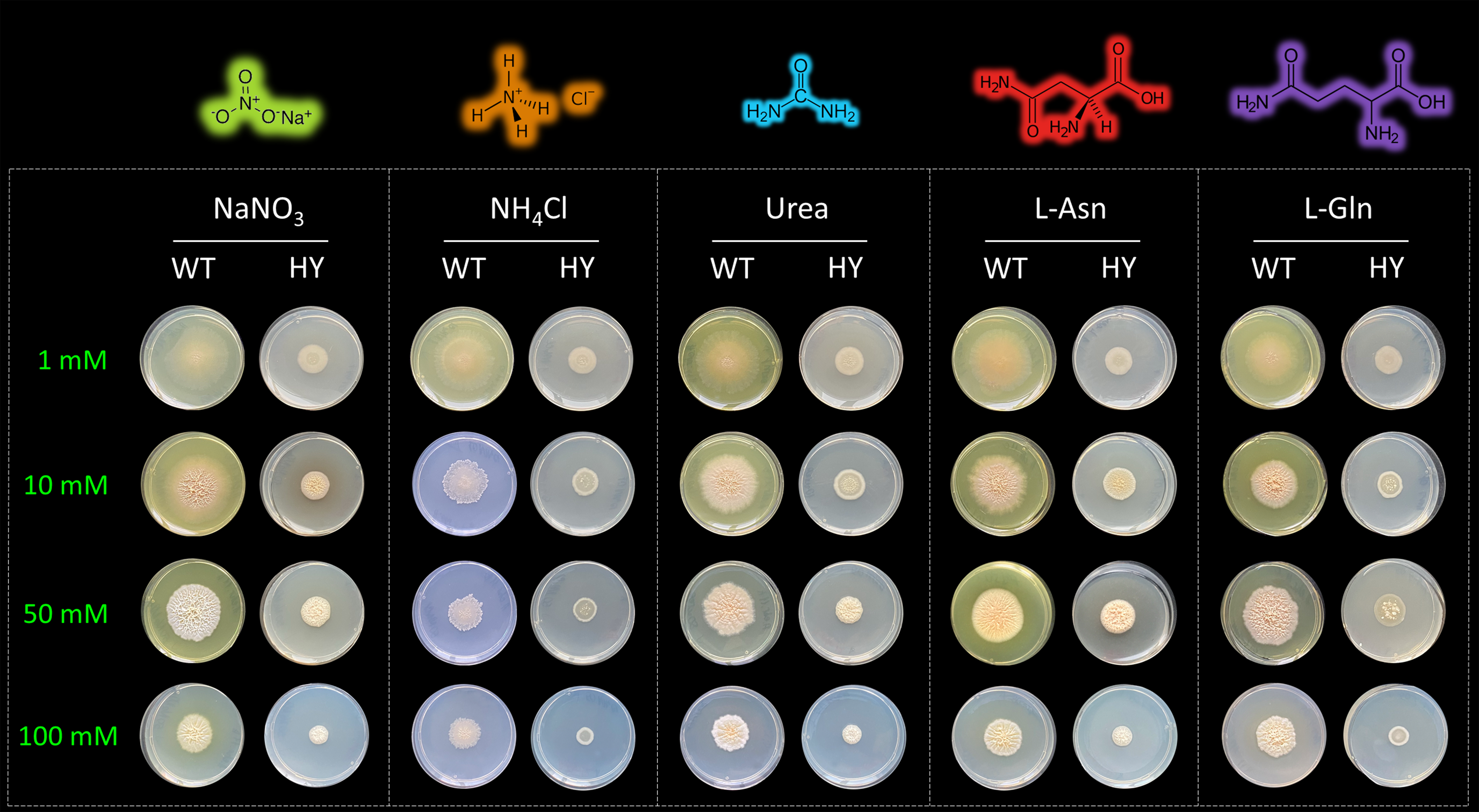

Supplement: Supplementary file 1 [file ijms-23-14625-s001.zip › S2 Figure.tif]

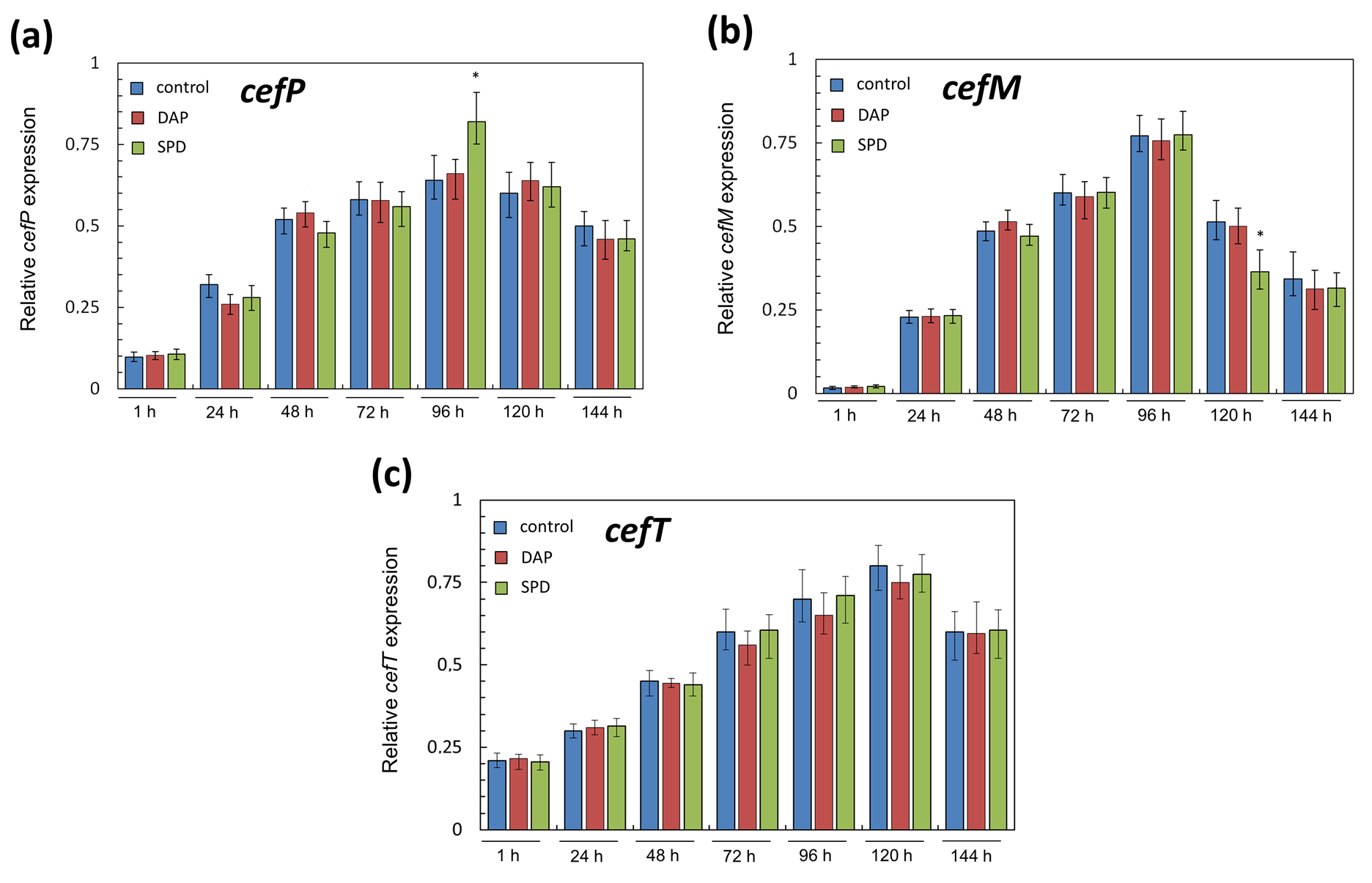

Supplement: Supplementary file 1 [file ijms-23-14625-s001.zip › S3 Figure.tif]
